# Understanding Lineage Plasticity as a Path to Targeted Therapy Failure in *EGFR*-Mutant Non-small Cell Lung Cancer

**DOI:** 10.3389/fgene.2020.00281

**Published:** 2020-03-27

**Authors:** Tatiana Shaurova, Letian Zhang, David W. Goodrich, Pamela A. Hershberger

**Affiliations:** Department of Pharmacology and Therapeutics, Roswell Park Comprehensive Cancer Center, Buffalo, NY, United States

**Keywords:** *EGFR* mutant lung cancer, EGFR tyrosine kinase inhibitors, acquired resistance, lineage plasticity, epithelial-mesenchymal transition, neuroendocrine transformation, Rb1

## Abstract

Somatic alterations in the epidermal growth factor receptor gene (*EGFR*) result in aberrant activation of kinase signaling and occur in ∼15% of non-small cell lung cancers (NSCLC). Patients diagnosed with *EGFR*-mutant NSCLC have good initial clinical response to EGFR tyrosine kinase inhibitors (EGFR TKIs), yet tumor recurrence is common and quick to develop. Mechanisms of acquired resistance to EGFR TKIs have been studied extensively over the past decade. Great progress has been made in understanding two major routes of therapeutic failure: additional genomic alterations in the *EGFR* gene and activation of alternative kinase signaling (so-called “bypass activation”). Several pharmacological agents aimed at overcoming these modes of EGFR TKI resistance are FDA-approved or under clinical development. Phenotypic transformation, a less common and less well understood mechanism of EGFR TKI resistance is yet to be addressed in the clinic. In the context of acquired EGFR TKI resistance, phenotypic transformation encompasses epithelial to mesenchymal transition (EMT), transformation of adenocarcinoma of the lung (LUAD) to squamous cell carcinoma (SCC) or small cell lung cancer (SCLC). SCLC transformation, or neuroendocrine differentiation, has been linked to inactivation of TP53 and RB1 signaling. However, the exact mechanism that permits lineage switching needs further investigation. Recent reports indicate that LUAD and SCLC have a common cell of origin, and that trans-differentiation occurs under the right conditions. Options for therapeutic targeting of *EGFR*-mutant SCLC are limited currently to conventional genotoxic chemotherapy. Similarly, the basis of EMT-associated resistance is not clear. EMT is a complex process that can be characterized by a spectrum of intermediate states with diverse expression of epithelial and mesenchymal factors. In the context of acquired resistance to EGFR TKIs, EMT frequently co-occurs with bypass activation, making it challenging to determine the exact contribution of EMT to therapeutic failure. Reversibility of EMT-associated resistance points toward its epigenetic origin, with additional adjustments, such as genetic alterations and bypass activation, occurring later during disease progression. This review will discuss the mechanistic basis for EGFR TKI resistance linked to phenotypic transformation, as well as challenges and opportunities in addressing this type of targeted therapy resistance in *EGFR*-mutant NSCLC.

## Introduction

### Lung Cancer

Every year lung cancer is estimated to claim more lives than colorectal, breast, and prostate cancers combined, making it the deadliest cancer worldwide (Didkowska et al., 2016). Selection of treatment strategies for lung cancer patients depends on the stage, histopathologic and molecular subtype of the tumor. Patients with early stage disease are likely to be treated with surgical resection with or without adjuvant chemo- and radiation therapies (Latimer and Mott, 2015). Over 50% of patients, however, present with advanced disease, when surgical intervention is no longer an option. For these patients, accurate classification of the tumor is particularly important for selection of appropriate therapy.

Lung cancer is a heterogeneous group of malignancies, encompassing two main histologic subtypes, non-small cell lung cancer (NSCLC) and small cell lung cancer (SCLC). SCLC is linked to heavy tobacco smoke exposure and comprises ∼20% of all lung cancer cases (Huang et al., 2015; Travis et al., 2015). Since these tumors express neuroendocrine markers, such as synaptophysin and chromogranin, they are thought to arise from neuroendocrine cells within the lung epithelium (Park et al., 2011; Osmani et al., 2018). At the genomic level, ∼10% of all SCLCs are mutant in *PTEN*, and ∼90% are mutant in both *RB1* and *TP53* tumor suppressor genes (Yokomizo et al., 1998; Beasley et al., 2003; Meuwissen et al., 2003; Sutherland et al., 2011). SCLC usually presents at late advanced stages and is seldom surgically resectable. Immune-based therapies in combination with systemic chemotherapy plays a critical role in management of advanced stage SCLC.

**FIGURE 1:**
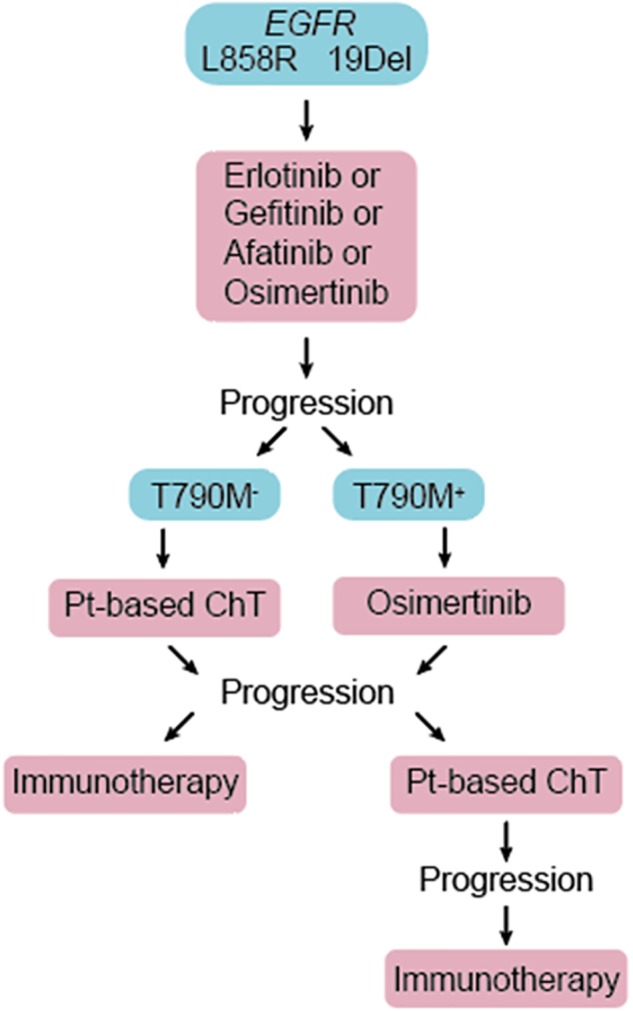
Patients diagnosed with advanced *EGFR*-mutant NSCLC are treated with one of the approved EGFR TKIs in the first line. Upon disease progression, repeat molecular testing is recommended. Patients not previously treated with osimertinib and whose tumor acquired second site T790M mutation in the *EGFR* gene (T790M^+^) are treated with 3rd generation EGFR TKI, osimertinib. Platinum-based chemotherapy (Pt-based ChT) and immunotherapies are reserved for T790M^–^ patients, and those who progress on osimertinib.

The majority of all lung cancers (80–85%) are NSCLC, of which adenocarcinoma of the lung (LUAD) is the most prevalent subtype (Latimer and Mott, 2015). Compared to SCLC, LUAD is less tightly associated with smoking (Couraud et al., 2012). Genetic alterations frequently seen in LUAD include mutations in the *TP53, KRAS*, and *EGFR* genes, as well as *ALK-NPM* fusion. LUAD lesions typically develop in the periphery of the lung and are usually diagnosed at a late advanced stage due to delayed occurrence of symptoms (Latimer and Mott, 2015). Before the era of precision medicine, treatment selection for advanced NSCLC was limited to systemic chemo-and radiation therapies. Recent development of immune checkpoint inhibitors and discoveries of targetable molecular drivers in NSCLC revolutionized the clinical care of these patients. Arguably, the most dramatic improvement in clinical outcomes are seen in patients whose tumors harbor one of several activating mutations in the epidermal growth factor receptor (*EGFR*) gene and are treated with small molecule EGFR tyrosine kinase inhibitors (EGFR TKIs) (Rosell et al., 2012; Sequist et al., 2013; Douillard et al., 2014; Mok et al., 2017; Soria et al., 2018). Immune-based therapies are approved for use in patients with no actionable molecular tumor alterations (Figure 1) but are not indicated for front-line use in *EGFR*-mutant NSCLC patients that are the subject of this review (Lee C. K. et al., 2017).

### EGFR as a Therapeutic Target in Lung Cancer

EGFR is a plasma membrane receptor tyrosine kinase that, upon ligand activation, auto phosphorylates and functions as a rapid activator of downstream signaling cascades (Ushiro and Cohen, 1980). The key targets of EGFR include mitogen-activated protein kinase (MAPK), phosphatidylinositol 3-kinase (PI3K/AKT), and Janus kinase (JAK)/signal transducer and activator of transcription (STAT) pathways (Bjorge et al., 1990; Zhong et al., 1994; Franke et al., 1995). The abovementioned transduction networks link EGFR tyrosine kinase activity to increased proliferation, motility, pro-survival, and anti-apoptotic cellular responses and highlight the oncogenic potential of abnormal EGFR activation.

The initial interest in exploiting EGFR as a principal therapeutic target was prompted by the results of molecular profiling studies that determined *EGFR* overexpression or amplification in 5–11% of LUAD (Cancer Genome Atlas Research Network, 2014; Gupta et al., 2009). First generation small molecule tyrosine kinase inhibitors, erlotinib (Tarceva) and gefitinib (Iressa), were developed and demonstrated significant survival improvement in advanced NSCLC patients who failed chemotherapy (Shepherd et al., 2005). Although early clinical trials demonstrated objective response rates to first generation EGFR TKIs below 10%, some patients experienced dramatic and durable response to these agents, pointing toward the presence of a unique prognostic feature in tumors that were exquisitely sensitive to EGFR inhibition (Fukuoka et al., 2003; Shepherd et al., 2005; Thatcher et al., 2005).

The subsequent discovery of somatic alterations in the tyrosine kinase domain of the *EGFR* gene provided an explanation as to why only a fraction of lung cancer patients had excellent response to EGFR TKIs (Shigematsu and Gazdar, 2006; Suzuki et al., 2005). The majority of tyrosine kinase domain mutations occur as deletions in exon 19 (Del19, ∼44%) and point mutations in exon 21 (L858R, ∼41%) (Shi et al., 2014; Kobayashi et al., 2015; Shigematsu et al., 2005). Mutant EGFR undergoes ligand-independent autophosphorylation to constitutively activate downstream oncogenic signaling (Okabe et al., 2007). These activating mutations confer reduced receptor affinity for ATP and allow EGFR TKIs to preferentially occupy the ATP-binding site of the receptor (Carey et al., 2006; Gazdar, 2009). Lower ATP binding affinity for the mutant receptor is the key to success of EGFR TKIs in the clinic, as it permits competitive EGFR inhibitors to achieve efficacious concentrations *in vivo* with a favorable toxicity profile (Shepherd et al., 2005). Not surprisingly, Del19- or L858R-positive NSCLC tumors are intrinsically sensitive to early generation EGFR targeting therapies. Today, several EGFR TKIs have gained an approval as standard of care in patients with advanced *EGFR*-mutant NSCLC. Hence, defining *EGFR* mutation status is a critical step in lung cancer diagnosis. Targetable *EGFR* mutations occur in ∼15% of NSCLC patients in the US and Europe, and ∼30% of NSCLC patients of Asian descent (Zhang et al., 2016).

### Acquired Resistance to EGFR TKIs

As *EGFR*-mutant tumors are “addicted” to oncogenic EGFR signaling (Politi et al., 2006), it is not surprising that persistent blockade of EGFR forces tumors to adapt and escape the anti-proliferative effects of EGFR TKIs. Therefore, the dramatic initial response to these agents is inevitably followed by emergence of treatment resistance. Generally speaking, mechanisms of acquired resistance to EGFR TKIs can be placed into one of three categories: EGFR-dependent resistance (50–60%), activation of bypass signaling (20–30%), and histologic or phenotypic transformation (5–10%) (Sequist et al., 2011; Yu et al., 2013).

EGFR-dependent resistance is marked by appearance of additional mutations in the *EGFR* gene. For instance, the T790M, or “gatekeeper” mutation, increases receptor binding affinity for ATP, resulting in loss of response to early generation TKIs (Yun et al., 2008). In a series of clinical trials, third generation EGFR TKI osimertinib demonstrated a favorable toxicity profile and 70% objective response rate in *EGFR*-mutation positive NSCLC patients whose disease progressed on early generation EGFR TKIs (Goss et al., 2016; Mok et al., 2017). Moreover, osimertinib was proven to be superior to first generation EGFR TKIs in the first line treatment setting (Soria et al., 2018). As with early generation EGFR targeting agents, acquired resistance to osimertinib is commonplace and can be attributed to third site mutations in *EGFR* (such as C797S), bypass activation, or phenotypic transformation (Kim et al., 2015; Thress et al., 2015; Ham et al., 2016; Ortiz-Cuaran et al., 2016; Li et al., 2017b; Oztan et al., 2017; Ricordel et al., 2017; Yang et al., 2018).

Activation of alternative tyrosine kinases (AXL, IGFR, FGFR, FAK, etc.) leads to loss of dependency on EGFR signaling and development of resistance to EGFR blockade (Guix et al., 2008; Kim et al., 2012; Zhang et al., 2012; Cortot et al., 2013; Ichihara et al., 2017; Lee A. F. et al., 2017). Many of these kinases are known to activate MAPK, AKT, or JAK/STAT networks, ultimately replacing, or bypassing, the requirement for pro-tumorigenic EGFR signaling. Ongoing clinical trials are investigating new targeted agents that inhibit these alternative kinases for their ability to overcome resistance to EGFR TKIs as mono-treatment modalities or in combination with approved EGFR therapeutics (ClinicalTrials.gov identifiers NCT02424617, NCT03450330, NCT03255083, NCT03123484). Phenotypic transformation can occur alongside or independently of bypass-linked resistance to EGFR TKIs. In the setting of acquired EGFR TKI resistance, phenotypic transformation encompasses EMT, SCLC, and transformation to squamous cell carcinoma (AST). In the remaining sections of this review, we will discuss in detail these less well understood mechanisms of EGFR TKI failure.

## Emt as a Mechanism of Resistance to Egfr Tkis

EMT is characterized by loss of intercellular adhesions and polarity, reorganization of cytoskeleton, and acquisition of invasive phenotype. At the molecular level, EMT is coordinated by increased expression of pro-mesenchymal transcription factors including Snail (*SNAIL*), Twist (*TWIST1*), and Zeb (*ZEB1/2*) families, and loss of a number of epithelial markers, including E-cadherin (*CDH1*), epithelial cell adhesion molecule (*EPCAM*), and claudin 4 (*CLDN4*) (De Craene and Berx, 2013). Loss of epithelial integrity facilitates tumor invasion of adjacent tissues, metastatic dissemination, and contributes to chemoresistance.

### The Role of Epithelial Phenotype in Intrinsic Sensitivity to EGFR TKIs

As previously discussed, early clinical trials of first generation EGFR TKIs were conducted in molecularly non-selected cohorts of patients with advanced NSCLC (Fukuoka et al., 2003; Shepherd et al., 2005; Thatcher et al., 2005). Dramatic tumor response to EGFR blockade was observed only in a small proportion of patients. These findings prompted a considerable research effort to determine prognostic tumor biomarkers of intrinsic sensitivity and resistance to EGFR targeted therapeutics. Several groups reported that an EMT gene expression signature is predictive of reduced EGFR TKI efficacy *in vitro* and *in vivo*. Using panels of predominantly *EGFR*-wild type NSCLC cell lines, several EGFR TKI response signatures were derived (Thomson et al., 2005; Yauch et al., 2005; Witta et al., 2006; Byers et al., 2013). Common to all proposed signatures, sensitivity to first generation EGFR TKIs was linked to high expression of a well-established epithelial marker *CDH1*, while resistance was associated with high expression of pro-mesenchymal markers vimentin (*VIM*) and *ZEB1*. The ability of these signatures to predict clinical response to EGFR TKIs was tested in cohorts that included both *EGFR*-mutant and *EGFR*-wild type NSCLC patients (Yauch et al., 2005; Kim et al., 2011). Although EGFR TKIs were more efficacious in patients whose tumors were classified as epithelial, it is important to note that *CDH1* expression since has been reported to be positively correlated with *EGFR* mutation status (Zhang et al., 2006; Zhao et al., 2015). Therefore, the apparent association between tumor epithelial phenotype and sensitivity to EGFR TKIs might have been driven by the presence of activating *EGFR* mutations in tumors with more epithelial phenotype.

The importance of EMT in predicting initial response to EGFR targeted therapies in the setting of *EGFR*-mutant NSCLC is still unclear. Several reports indicate a trend toward better objective response rate in patients treated with first generation EGFR TKIs whose tumors express high levels of E-cadherin and can be classified as epithelial, but the results do not reach statistical significance (Chen et al., 2013; Ren et al., 2014).

### The Role of EMT in Acquired Resistance to EGFR TKIs

#### EMT Co-occurring With Bypass Kinase Activation

EMT is reported in a number of pre-clinical studies that investigate acquired resistance to EGFR TKIs. However, it is difficult to determine whether EMT is the sole driver of resistance in every case, since it frequently occurs alongside bypass activation. The most common indicator of bypass-associated EGFR TKI resistance is loss of TKI-induced phosphorylation blockade at the main downstream targets of EGFR, such as MAPK and AKT, in the absence of additional mutations in the *EGFR* gene. MAPK and AKT are critically located at the interface of multiple oncogenic signaling cascades reported to be activated as a part of EGFR TKI resistance. Therefore, alternative kinase activation is likely to involve one or both of these proteins. Recently, Src/FAK emerged as a critical node in the EGFR bypass network.

Src/FAK are non-receptor tyrosine kinases that can be activated by EGFR and multiple other plasma membrane RTKs, including AXL, IGF1R, and FGFR (Sandilands et al., 2007; Sulzmaier et al., 2014; Cox et al., 2015; Marlowe et al., 2016). The Src/FAK network converges on AKT and MAPK to sustain oncogenic signaling and induce EMT. Many of the RTK that signal through Src/FAK have been implicated in EMT-associated resistance to EGFR targeting agents (Tang et al., 2016; Ichihara et al., 2017; Nukaga et al., 2017; Weng et al., 2018).

Activation of AXL tyrosine kinase was one of the first identified bypass-associated mechanisms of resistance to EGFR TKIs (Zhang et al., 2012; Okimoto and Bivona, 2015). AXL is a member of TAM family of receptors that is capable of activating AKT and MAPK signaling cascades. Hence, in the setting of persistent blockade of the EGFR pathway, AXL may play a critical role in cell survival, providing an alternative axis of pro-tumorigenic signal propagation. Moreover, AXL is known to govern cytoskeletal rearrangement and invasive capacity in lung cancer (Shieh et al., 2005). Early reports indicated that in murine xenograft- and cell line-based models of acquired resistance to first generation EGFR TKIs, *AXL* was among the most upregulated genes compared to control, erlotinib-naïve specimens (Zhang et al., 2012). Strikingly, resistance was also accompanied by a dramatic upregulation of *VIM* and near complete loss of *CDH1* expression. Inhibition of AXL kinase activity restored both *CDH1* expression and sensitivity to EGFR TKIs. Overexpression of *AXL* is also linked to EMT-associated resistance to 3rd generation EGFR TKI, osimertinib (McGowan et al., 2017). Therefore, AXL remains a promising next line therapeutic target for EMT-associated resistance, with several pharmacological inhibitors in early clinical development (ClinicalTrials.gov identifiers NCT03255083, NCT02424617, NCT02729298).

Activation of Insulin-like growth factor 1 receptor (IGF1R) as an alternative to EGFR signaling can also occur as a part of EMT-linked resistance. Similar to EGFR, IGF1R signal transduction primarily involves MAPK and AKT pathways and may lead to mesenchymal transdifferentiation (Vazquez-Martin et al., 2013). Elevated levels of IGF1R have been documented in pre-clinical models of acquired resistance to EGFR TKIs, where its expression inversely correlates with epithelial phenotype (Vazquez-Martin et al., 2013; Zhou et al., 2015; Hussmann et al., 2017; Li et al., 2017a). Intriguingly, tumor IGF1R expression is associated with lymphatic invasion and predicts shorter progression-free survival in *EGFR*-mutant but not *EGFR*–wild type NSCLC, suggesting a unique interplay between EGFR and IGF1R pathways in this type of malignancy (Park et al., 2015). The above observations point toward therapeutic relevance of IGF1R. However, the results of early clinical trials of anti–IGF-1R monoclonal antibodies R1507 or figitumumab in combination with erlotinib demonstrated unfavorable toxicity profiles and lack of improved response (Ramalingam et al., 2011; Belani et al., 2012). It is important to note that the abovementioned clinical trials were conducted in molecularly non-selected cohorts of lung cancer patients. Therefore, it is possible that IGF1R remains an important target in *EGFR*-mutant NSCLC.

To compensate for consistent EGFR blockade, some tumor cells activate fibroblast growth factor family of receptors (FGFR). FGFR activation promotes mesenchymal transdifferentiation and reduces sensitivity to EGFR TKIs. Indeed, it appears that in the context of acquired resistance to EGFR inhibitors, FGFR activation is almost inevitably accompanied by gain of mesenchymal features (by phenotypic transformation toward more mesenchymal phenotype) (Jakobsen et al., 2017). To address causative relationship between FGFR1 activation and onset of EMT, either *FGFR1* or *ZEB1* were overexpressed in *EGFR*-mutant, treatment-naïve cells (Vad-Nielsen et al., 2019). Cells expressing high levels of *ZEB1* underwent EMT and upregulated *FGFR1* expression. Surprisingly, no induction of EMT was noted in cells overexpressing *FGFR1*. These observations suggest that the FGFR1-associated kinase switch occurs as a consequence of EMT and not the other way around. Nevertheless, targeted inhibition of FGFR may be a viable strategy to overcome resistance to EGFR TKIs linked to EMT (Raoof et al., 2019).

#### EMT in the Absence of Bypass Activation

EMT as the sole driver of acquired resistance to EGFR TKIs was initially identified in a series of clinical case studies (Uramoto et al., 2010; Chung et al., 2011; Sequist et al., 2011; Uramoto et al., 2011). The first report of a trend toward EMT in *EGFR*-mutant NSCLC with acquired resistance to EGFR TKI gefitinib came in 2010, when biospecimens from 9 cases of *EGFR*-mutant lung adenocarcinoma pre- and post-acquisition of resistance to gefitinib were analyzed for expression of epithelial (E-cadherin, γ-catenin) and mesenchymal (vimentin, fibronectin) markers (Uramoto et al., 2010). Although statistical significance was not achieved, possibly due to small sample size, the report indicated that loss of E-cadherin and gain of mesenchymal markers may be associated not only with intrinsic, but also with acquired resistance to EGFR targeting agents. Results from a larger study suggested that EMT may be a relatively rare mechanism of EGFR TKI failure, affecting ∼5% of patients (Sequist et al., 2011). EMT occurred in the absence of any other mechanisms of resistance known at the time, and therefore was determined to be the basis for therapeutic failure. It is important to note that early studies investigated the most common mechanisms of resistance known at the time, mainly involving additional genomic alterations in the *EGFR* gene and several other known oncogenic drivers, such as amplification of *MET* and *PIK3CA* mutations. Therefore, activation of an alternative tyrosine kinase might have been easily missed.

Nevertheless, involvement of EMT in acquired resistance to EGFR TKIs is supported by a number of studies conducted in pre-clinical models of *EGFR*-mutant NSCLC. Many of them, as discussed earlier, suggested that transition toward mesenchymal phenotype co-occurs with a kinase switch. However, in some cases, bypass activation was not identified, suggesting that EMT itself might be responsible for the development of therapy-refractory disease (Lee A. F. et al., 2017; Song et al., 2018; Yochum et al., 2019).

The mechanistic basis for the role of E-cadherin in the efficacy to EGFR TKIs may be placed in its ability to activate cell-intrinsic apoptosis. Cell intrinsic apoptosis is initiated by binding of tumor necrosis factor family ligands, such as Apo2L/TRAIL to death receptors, TRAILR1/2. This binding event ultimately leads to assembly of the death-inducing signaling complex and caspase-8 activation (Wilson et al., 2009). E-cadherin has been shown to physically interact with ligated TRAIL1/2, couple them to the actin cytoskeleton, and promote activation of cellular caspases. Intriguingly, *EGFR*-mutant cells with acquired EMT-associated resistance to erlotinib develop resistance to Apo2L (Lu et al., 2014).

Although a link between intrinsic sensitivity to EGFR TKIs and tumor E-cadherin expression has been proposed, the direct role of E-cadherin loss in acquisition of resistance to this class of drugs is not universally agreed upon. A dramatic reduction in E-cadherin levels is a common feature of EMT-associated EGFR TKI resistance. However, shRNA –dependent depletion of *CDH1* in *EGFR*-mutant therapy-naïve NSCLC cells does not necessarily lead to loss of EGFR TKI efficacy (Lee A. F. et al., 2017). It is possible that, in addition to low expression of epithelial molecules, upregulation of mesenchymal markers is necessary to drive EGFR TKI resistance.

Ectopic expression of the Snail family of transcription factors represses *CDH1* expression to induce EMT and resistance to EGFR TKIs in *EGFR*-mutant NSCLC (Lee A. F. et al., 2017). Additionally, expression of pro-mesenchymal factor TWIST in *EGFR*-mutant NSCLC has been linked to induction of EMT and acquired resistance to EGFR TKIs. Some studies suggest that TWIST can inhibit transcriptional activation of pro-apoptotic protein BIM either by directly binding to its promoter, or by inducing *ZEB1*, which then acts as a repressor of BIM transcription (Song et al., 2018; Yochum et al., 2019). Therefore, BH3 mimetics, a class of drugs that target BCL family of proteins to promote apoptosis, may be beneficial for treating EMT-associated EGFR TKI resistance. Navitoclax, a novel BH3 mimetic compound, is currently under early phase clinical investigation in *EGFR*-mutation positive NSCLC patients who relapsed on previous EGFR TKI therapy (CilicalTrials.gov identifier NCT02520778).

Activation of Notch-1 has also been linked to EMT-associated EGFR TKI resistance. In cell-based and murine models, acquired resistance to gefitinib was accompanied not only by increased expression of mesenchymal markers and diminished E-cadherin, but also upregulation of Notch-1 (Xie et al., 2012, 2013). Notch-1 silencing resulted in E-cadherin re-expression and partial restoration of sensitivity to gefitinib. Pharmacological inhibition of Notch-1 signaling pathway is currently in early clinical development (NCT01158404, NCT01653470).

#### The Use of Epigenetic Agents in the Context of Acquired Resistance to EGFR TKIs

Development of drug “tolerance,” a reversible state of reduced sensitivity to treatment associated with mesenchymal phenotype, precedes the onset of more permanent resistance (Sharma et al., 2010). Drug tolerant persisters (DTPs) harbor an altered chromatin state with high expression levels of KDM5A, a H3K4 histone demethylase, and other chromatin-modifying enzymes. Therefore, the use of epigenetic agents may be beneficial to combat phenotypic plasticity as part of acquired drug resistance. Indeed, several histone deacetylate inhibitors (HDACi), including entinostat and trichostatin A, have been shown to restore expression of *CDH1* and promote sensitivity to EGFR TKIs in pre-clinical models of *EGFR*-mutant NSCLC (Sharma et al., 2010; Suda et al., 2011; Weng et al., 2018).

Combination of HDACi vorinostat and erlotinib was investigated in a phase I/II clinical trial, where it demonstrated a favorable toxicity profile but failed to provide survival benefit in *EGFR*-mutant NSCLC patients with prior progression on erlotinib (Reguart et al., 2014). However, out of 19 patients included in the phase II part of the study, 7 were positive for the T790M mutation in the *EGFR* gene and therefore were unlikely to derive any benefit from erlotinib-based combination treatment. It is worth noting, that a set of patients achieved long-term stable disease on the vorinostat/erlotinib regimen. No additional molecular profiling was performed to pinpoint the features associated with susceptible tumors. In a larger phase II trial conducted in molecularly non-selected advanced stage NSCLC, combination of erlotinib and entinostat produced a significant survival benefit only in patients with high tumor E-cadherin expression (Witta et al., 2012). Further investigation of epigenetic modifiers in combination with contemporary 3rd generation EGFR TKI osimertinib in *EGFR*-mutant NSCLC are needed to determine the utility of epigenetic drugs in the context of acquired resistance to EGFR targeted therapeutics that is associated with phenotypic transformation.

#### Challenges in Addressing EMT-Associated Resistance to EGFR TKIs

EMT emerges with acquired resistance to EGFR TKIs. However, the direct cause and effect relationship between EMT and EGFR TKI resistance appears to be context dependent. The majority of pre-clinical reports that link loss of sensitivity to anti-EGFR drugs to mesenchymal phenotype demonstrate the co-occurrence of a kinase switch. Hence, it is a challenging to determine whether EMT is truly a driver of therapeutic failure or simply a passenger to some other primary mechanism of resistance (i.e., bypass activation). The notion that epithelial phenotype directly correlates with the efficacy of EGFR targeted therapies was put forward when early clinical trials performed in non-selected NSCLC patients identified a link between tumor E-cadherin levels and response to first generation EGFR TKI erlotinib (Richardson et al., 2012). The research was focused on identifying EMT signatures that could predict sensitivity to early generation TKIs in patients with undefined *EGFR* mutation status. As a result, epithelial phenotype in general, and E-cadherin expression specifically, were determined to be important contributors to intrinsic sensitivity to EGFR TKIs. However, current knowledge dictates that activating mutations within tyrosine kinase domain of the EGFR predict clinical relevance of TKIs, and their use remains limited to the *EGFR*-mutant subset of NSCLC. Moreover, EGFR signaling appears to be critical for epithelial lineage differentiation and it is now clear that *EGFR*-mutant tumors are more likely to express high levels of E-cadherin than tumors with wild type receptor (Miettinen et al., 1995). Therefore, in the context of molecularly non-selected patients and cell lines, the sensitivity to EGFR TKIs may be driven not by the epithelial phenotype *per se*, but rather by the positive *EGFR* mutation status that is linked to such phenotype. Consequently, it remains unclear whether the EMT signatures derived from cell panels that included both *EGFR*-wild type and -mutant cell lines and validated in predominantly *EGFR*-wild type clinical cohorts are optimal for the study of EMT as a basis for acquired resistance to EGFR TKIs in *EGFR*-mutant NSCLC.

EMT is characterized as a complex multistep process that involves molecular changes that are often context dependent. Therefore, a pleotropic agent that is capable of initiating a broad-spectrum epithelial differentiation program could be of exceptional therapeutic value. We previously reported that 1,25-dihydroxyvitamin D3 [1,25(OH)2D3], an active metabolite of vitamin D, is uniquely suitable for applications in EGFR-mutant NSCLC (Upadhyay et al., 2013). Vitamin D receptor (VDR), a critical player in vitamin D signaling axis, is highly expressed in *EGFR*-mutant lung cancer cells, making them intrinsically sensitive to 1,25(OH)2D3. Moreover, 1,25(OH)2D3 restores expression of E-cadherin in models of EMT-associated resistance to EGFR TKIs, suggesting its capacity to promote sensitivity to this class of therapeutics (Liu et al., 2018). Studies to elucidate clinical relevance of 1,25(OH)2D3-based combination therapies in the context of acquired resistance to EGFR TKIs are currently under way in our laboratory.

To effectively address EMT-associated resistance, its onset must be reliably detected in clinical samples. Traditional or liquid biopsy is commonly performed in *EGFR*-mutation positive NSCLC patients who failed first line EGFR TKI therapy. Generally, contemporary tests allow for detection of additional mutations in the *EGFR* and genomic alterations in a limited number of resistance-associated genes, such as amplification of MET. However, EMT as a potential mechanism of resistance is not routinely investigated. Lack of a clinically validated panel of biomarkers that identify the transition toward mesenchymal phenotype in tumor biopsies and in circulating tumor cells, is a hurdle yet to be overcome.

**FIGURE 2:**
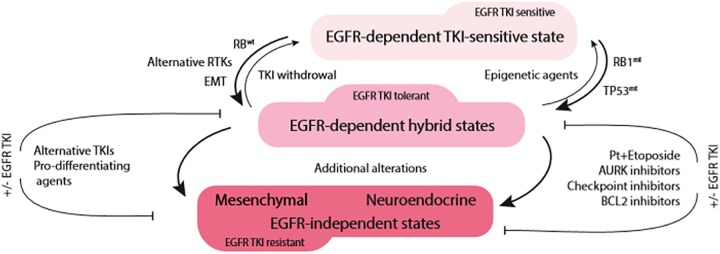
Acute EGFR TKI exposure drives phenotypic plasticity and TKI tolerance in *EGFR*-mutant NSCLC. The route to therapeutic resistance appears to be dependent on RB1 status. In RB1-wild type tumors (RB1wt), transition toward mesenchymal phenotype with activation of alternative tyrosine kinases (RTKs) is likely to occur (left side of the diagram). On the other hand, RB1-mutant/TP53-mutant (*RB1*mt, *TP53*mt) tumors are likely to undergo neuroendocrine transformation (right side of the diagram). This hybrid, EGFR TKI-tolerant state appears to be still dependent on EGFR signaling and may revert to EGFR TKI-sensitive phenotype upon EGFR TKI withdrawal or epigenetic therapies. With prolonged treatment, due to additional genomic alterations, tumors lose their dependence on EGFR signaling and lock in the terminal state of EGFR TKI resistance.

The frequency of EMT as a part of acquired resistance to EGFR TKIs may be greatly underestimated not only in the clinic, but also at the laboratory bench. In many cases, primary investigation into the mechanisms underlying therapeutic failure is aimed at determining acquisition of genetic alterations in the *EGFR* gene and activation of alternative tyrosine kinases. Often, phenotypic transformation as a factor contributing to loss of sensitivity is considered only in the absence of the abovementioned mechanisms. However, it is becoming abundantly clear that EMT may occur concomitantly with activation of bypass signaling. Therefore, whether EMT is a driver of the resistance or a passenger to the primary mechanism, i.e., bypass activation, may be difficult to establish. It is possible that the phenotypic state of the tumor cells dictates dependency on a particular set of growth factors. Thus, while epithelial cells depend on EGFR signaling, cells that undergo EMT may depend instead upon RTKs such as AXL, IGF1R, or FGFR, while EGFR signaling becomes less relevant. In this context, the abovementioned RTKs not only allow the cells to retain mesenchymal properties but also serve as markers of the adoption of an alternative phenotypic state. For instance, similar to EGFR TKI-resistant tumor cells, normal lung fibroblasts express high levels of FGFR and PDGFR, indicating the critical importance of these growth factor signals in survival of mesenchymal cells (Green et al., 2016; Danopoulos et al., 2019). Therefore, in contrast to the conventional paradigm that separates EMT-associated resistance to EGFR TKIs from bypass-dependent disease, these phenomena can be united as two sides of a phenotypic switch driven by chronic EGFR inhibition (Figure 2). The implication of such a model is that EMT-associated resistance may be targeted either by using epigenetic drugs [or 1,25(OH)2D3] to maintain/restore epithelial phenotype and EGFR TKI sensitivity or inhibiting the alternative receptor dependencies of mesenchymal variants.

## Phenotypic Transformation to Sclc as a Mechanism of Resistance to Egfr Tkis

### SCLC Is Diagnosed in a Subset of EGFR-Mutant Patients at the Time of EGFR TKI Failure

The transformation of *EGFR*-mutant LUAD to SCLC (referred to as SCLC transformation or lineage transformation) accounts for up to 14% of acquired resistance to EGFR TKIs (Sequist et al., 2011). Pre-clinical models that faithfully recapitulate SCLC transformation have yet to be developed. Thus, our understanding of this important mechanism of resistance to date has come exclusively from the analysis of clinical specimens. In an early case report, a 45-year-old female patient who never smoked was diagnosed with LUAD and treated with erlotinib (Zakowski et al., 2006). She responded well for 18 months, but then her disease progressed. A biopsy performed at the time of relapse showed evidence of SCLC. SCLC was identified by morphology and confirmed with immunohistochemical staining for synaptophysin. Typically, SCLC cells are positive for neuroendocrine markers including synaptophysin, chromogranin, or CD56. Since this initial observation, numerous additional cases have been reported in which SCLC is absent in initial diagnostic samples of *EGFR*-mutant LUAD but detected at the time of EGFR TKI failure (Oser et al., 2015). Therapy associated SCLCs have reduced expression of EGFR protein compared to corresponding baseline tumors (Niederst et al., 2015), which could partially explain why they fail to respond to EGFR TKIs (Zakowski et al., 2006; Popat et al., 2013). Treatment of therapy associated SCLC follows classical SCLC guidelines, typically a combination of platinum and etoposide (Ham et al., 2016).

### EGFR TKI Resistant SCLC Arises From the Original EGFR-Mutant LUAD

In theory, SCLC detected at the time of EGFR TKI failure could arise *de novo* or via transformation of the original *EGFR*-mutant LUAD. Two complementary lines of evidence support the latter mechanism. First, SCLC detected at relapse harbors the same *EGFR* mutations as the original LUAD, indicating a shared clonal origin (Niederst et al., 2015; Lee J. K. et al., 2017). Second, *EGFR* activating mutations are rarely detected in *de novo* SCLC (frequency ranges from 0 to 4% in different case series). Interestingly, SCLC is also observed at the time of relapse in a subset of patients diagnosed with *ALK*-positive LUAD who receive targeted therapy with ALK TKIs (Levacq et al., 2016). However, SCLC is not observed in patients with *EGFR* wild-type LUAD that develop resistance to chemotherapy (Sequist et al., 2011). Cumulatively, these observations suggest that SCLC transformation is not a generic mechanism of therapeutic resistance. Rather, tumor transformation occurs in response to targeted inhibition of oncogenic drivers. As improved molecularly targeted therapies are integrated into clinical practice, the frequency of SCLC transformation is predicted to rise. This highlights the need to understand the molecular mediators of treatment associated SCLC transformation so that appropriate interventions can be developed.

### Molecular Predictors of SCLC Transformation

To identify candidate mediators of SCLC transformation, Niederst et al. used genomic sequencing to compare the molecular profiles of EGFR TKI resistant-NSCLC and EGFR TKI resistant-SCLC samples that were obtained from a single patient who was initially diagnosed with *EGFR*-mutant LUAD (Niederst et al., 2015). The SCLC transformed samples contained the original *EGFR* mutation plus additional mutations in *TP53* and *PIK3CA*. SCLC transformed tumors also uniquely showed evidence of *RB1* loss. Subsequent immunohistochemistry assays performed on 10 additional cases of transformed SCLC demonstrated that loss of RB1 protein (pRb) is ubiquitous. Notably, *TP53* and *RB1* mutation are also ubiquitous in classical SCLC. Thus, transformed SCLC recapitulates the molecular features of classical SCLC. In contrast, pRb was retained in 10/11 *EGFR*-mutant LUADs that developed EGFR TKI resistance without SCLC transformation. These data implicate *RB1* loss as a driver of SCLC transformation.

A causal role for *RB1* is further supported by the observation that patients who present with *EGFR/TP53/RB1* triple mutant lung cancers (approximately 5% of all *EGFR*-mutant cases) are at a sixfold higher risk for SCLC transformation compared to patients with *EGFR*-mutant lung cancer who do not harbor concurrent alterations in *TP53* and *RB1* (Offin et al., 2019). However, it is important to acknowledge that not all *EGFR/TP53/RB1* triple mutant lung cancers transform as disease progresses. We can infer from these data that *TP53/RB1* mutation may be necessary, but is not sufficient, for SCLC transformation. Consistent with this idea, *RB1* knockdown in *EGFR*-mutant LUAD cell lines is also not sufficient for transdifferentiation into SCLC (Niederst et al., 2015).

An existing mouse model of classical SCLC provides some insight into other factors that may be required. In this model, *TP53* and *RB1* were inactivated in pulmonary epithelial cells (Meuwissen et al., 2003). Aggressive lung tumors developed. Cell lines developed from resultant tumors displayed mixed (neuroendocrine and non-neuroendocrine) phenotypes. Molecular characterization of these cell lines showed that neuroendocrine differentiation was observed only in cells that also harbor amplification of *LMYC* and increased expression of Ascl-1 (Calbo et al., 2005). The requirement for multiple genetic hits to generate SCLC/neuroendocrine tumors may explain, at least in part, why pre-clinical models of *EGFR*-mutant transformed SCLC are still lacking.

### Role of the RB1 Encoded Protein (pRb) in SCLC Transformation

How might *RB1* loss facilitate SCLC transformation? *RB1* was the first tumor suppressor gene to be discovered, molecularly isolated in 1986 as the gene responsible for both hereditary and sporadic forms of the childhood cancer retinoblastoma. The canonical function of the *RB1* encoded protein (pRb) is to enforce cell cycle arrest in response to anti-proliferative signals. pRb mediates cell cycle control through its physical interaction with E2F transcription factors, recruiting chromatin modifying factors to repress transcription of E2F target genes. pRb activity is negatively regulated by cyclin dependent kinase (CDK) phosphorylation which inhibits pRb/E2F interaction leading to de-repression of a highly conserved gene expression program encompassing factors critical for cell cycle progression. It has become apparent in recent years that pRb has functions that extend beyond cell cycle control (Knudsen et al., 2019). Pertinent to the topic of SCLC transformation is the discovery that pRb suppresses the expression of pluripotency gene networks. *RB1* deletion in mouse and human fibroblasts increases the efficiency of induced pluripotent stem cell reprogramming (Kareta et al., 2015). It appears to involve de-repression of pluripotency regulatory factors like SOX2 and OCT4. We reasoned that *RB1* loss in the context of cancer would have similar effects. Indeed, we found that *RB1* deletion in mouse and human experimental models of prostate cancer also facilitated lineage plasticity with transformation of prostate adenocarcinoma to neuroendocrine (small cell) variants. This neuroendocrine transformation is accompanied by acquired resistance to androgen receptor targeted therapy. Lineage transformation and therapeutic resistance in our prostate cancer models were dependent on increased expression of stem cell reprogramming factors like SOX2 and EZH2. In an analogous manner, *RB1* loss may mediate upregulation of epigenetic and stem cell reprogramming factors in *EGFR*-mutant LUAD, facilitating SCLC transformation and acquired EGFR TKI resistance. Because reprogramming is reversible (at least until the phenotypic switch becomes genetically fixed), an opportunity may exist to combat SCLC transformation by therapeutic intervention with epigenetic modulating therapy.

### Potential Treatment Strategies for Those at Risk or Diagnosed With EGFR-TKI Therapy Associated SCLC

#### Use of Bcl-2 Inhibitors

Currently, *TP53* and *RB1* status does not inform clinical decision making for patients diagnosed with advanced *EGFR*-mutant NSCLC. Alteration of *RB1* and/or *TP53* is likely to increase the likelihood that *EGFR*-mutant adenocarcinoma will relapse as an SCLC variant. These variants have distinct therapeutic sensitivities and are generally lethal because they rapidly develop resistance (Offin et al., 2019). Thus, routine determination of *RB1/TP53* mutation status merits consideration, especially if therapeutic intervention can prevent or delay SCLC transformation. Once such at-risk patients are identified, several therapeutic options might be considered. In patients who present with triple mutant cancers, time to treatment failure could be extended by combining EGFR TKIs upfront with conventional SCLC therapy (platinum/etoposide). As suggested by Offin and colleagues, this combination is expected to be effective because EGFR TKIs will eradicate the bulk of *EGFR*-mutant LUAD cells. Platinum/etoposide chemotherapy will inhibit *EGFR/TP53/RB1* triple mutant subclones that escape EGFR TKI treatment. Clinical trials have been initiated to test this approach (NCT03567642). It is worth noting here that if platinum/etoposide is not given upfront but instead is used after SCLC transformation, a response rate of 54% is obtained (Marcoux et al., 2019). However, response is not durable (progression free survival under 4 months) (Marcoux et al., 2019). In this situation, Bcl-2 family inhibitors might be used to block progression. ABT-263, an orally bioavailable Bcl-2 family protein inhibitor, is effective in SCLC models (Shoemaker et al., 2008). *EGFR*-mutant, transformed SCLC cells are more sensitive to ABT-263 than EGFR-TKI-resistant NSCLC cell lines (Niederst et al., 2015). However, clinical trials on transformed SCLC patients will be needed to test its safety and efficacy.

#### Targeting Unique Cell Cycle Vulnerabilities Created by RB1 Loss

Another potential therapeutic option to counter the emergence of transformed SCLC is to target cell cycle vulnerabilities that are created upon *RB1* loss. In triple negative breast cancers (lack estrogen receptor, progesterone receptor, and Her2), *RB1* loss places stress on the DNA replication machinery, which results in sensitivity to CHK1 and PLK1 inhibitors that target DNA damage checkpoints (Witkiewicz et al., 2018). It is possible that *RB1* loss in *EGFR* mutant lung cancer cells will yield a similar, synthetically lethal interaction with these inhibitors.

Recent studies implicate Aurora kinases (AURKA or AURKB) that regulate mitotic spindle assembly and chromosome segregation as alternative therapeutic targets. Independent CRISPR/Cas9 (Oser et al., 2019) and drug screening assays (Gong et al., 2019) using *Rb*1-deficient classical SCLC cell lines simultaneously identified a unique dependence upon AURK for survival. In these studies, AURK inhibitors potently suppressed the growth of *Rb1*-negative SCLC cells but had no effect on *Rb1*-positive lung cancer cells (including the *EGFR* mutant cell lines H1975 and PC9). One might conclude based on these results that single agent AURK inhibitors will be useful in targeting the subpopulation of *EGFR* mutant cells that lack *Rb1* and are prone to SCLC transformation. However, the utility of AURKA inhibitors in *EGFR* mutant lung cancer exceeds this specific molecular context. This is because in *Rb1*-proficient models (like PC9 and H1975), third generation EGFR TKIs induce AURKA activation (Shah et al., 2019). Upon induction, AURKA blocks EGFR TKI-induced apoptosis and promotes drug resistance. In this scenario, AURKA inhibitors can be used in combination with EGFR TKIs to counter the emergence of drug resistance. AURK inhibitors thus hold considerable promise for prolonging the efficacy of third generation EGFR TKIs via their ability to target both *Rb1*-deficient clones that are prone to SCLC transformation *and* bulk *Rb1*-proficient cells that upregulate AURKA during EGFR TKI treatment. A Phase 1/1b clinical trial of AURKA inhibitor Alisertib with osimertinib in metastatic *EGFR*-mutant lung cancer is currently recruiting participants (NCT04085315).

#### Use of Epigenetic Therapies

A third approach to block SCLC transformation involves the application of epigenetic therapy. As discussed above, *RB1* loss promotes lineage transformation in prostate cancer via the upregulation of epigenetic and reprogramming factors including EZH2 (Ku et al., 2017). Pharmacologic inhibitors of EZH2 are currently being evaluated as cancer therapies, often in combination with other therapies (NCT03460977, NCT03525795, NCT04104776). We have determined that EZH2 inhibitors can suppress prostate cancer lineage plasticity, neuroendocrine transformation, and resistance to androgen receptor targeted therapies in pre-clinical mouse and human experimental models (Ku et al., 2017). We anticipate analogous results may be obtained in *EGFR*-mutant lung cancers that lose *RB1* since these variants are likely to up regulate EZH2 (Coe et al., 2013; Ishak et al., 2016). Supporting this idea, classical SCLCs have been found to overexpress EZH2 protein (Byers et al., 2012). Although it is conceivable that EZH2 inhibitors may reverse lineage transformation, based on pre-clinical experimental models, in the clinic it may be most beneficial to use them upfront in combination with EGFR-TKIs to prevent SCLC transformation, particularly in *EGFR/TP53/RB1* triple mutant lung cancers. The identity of other epigenetic factors that become dysregulated in *EGFR/TP53/RB1* mutant lung cancers and their effects on mutant *EGFR* lung cancer lineage plasticity and therapeutic resistance is an area of active research that may yield additional therapeutic targets.

#### Targeting New RTK Dependencies in SCLC Variants

Analogous to mesenchymal variants of *EGFR* mutant lung cancer, transformed SCLC variants may possess novel RTK dependencies that can be therapeutically targeted. Matsumura and colleagues found that c-Kit was the only RTK that is significantly expressed in high-grade neuroendocrine carcinomas of the lung, including both large cell neuroendocrine carcinoma (LCNEC) and SCLCs, but not in NSCLC (Matsumura et al., 2015). IGF1R and KDR also showed higher expression scores in SCLC and LCNEC than in LUAD. *FGFR1* amplification is also reported in SCLC and potentially may be used as a therapeutic target (Peifer et al., 2012). Another large group of tyrosine kinase receptors is vascular endothelial growth factor receptor (VEGFR) family. Many neuroendocrine tumors are hyper-vascular and histologically show a rich, well-developed vascular network. *VEGF-A* has been demonstrated to play a critical role in tumorigenesis and neuroendocrine tumor progression. Sunitinib can irreversibly inhibit VEGFR family and is effective in several solid tumors and pancreatic neuroendocrine tumors (Capozzi et al., 2016). It is possible that during the process of acquired EGFR TKI resistance, *RB1* loss could facilitate a shift in the signaling pathways activated, like the FGFR1 pathway, reducing dependency on EGFR. This shift in signaling pathway activation may, in turn, contribute to SCLC transformation. Many knowledge gaps will need to be filled to identify and rationally implement such approaches, but this is made difficult by the dearth of experimental models mimicking treatment associated SCLC transformation.

## Phenotypic Transformation to Scc as a Mechanism of Resistance to Egfr Tkis

Approximately one percent of *EGFR*-mutant lung cancer patients develop resistance to EGFR targeting therapeutics that is accompanied by a phenotypic switch from ADC to SCC (Clery et al., 2017; Park et al., 2019). Although SCC is a distinct histological subtype of NSCLC, lung tumors, including those positive for activating mutations in the *EGFR* gene with mixed adenosquamous histology, are known to occur (Ricciuti et al., 2018). Hence, some tumors may possess high potential for AST when certain genomic alterations are acquired. ADC to SCC lineage transformation remains the least common in the setting of acquired resistance to EGFR TKIs, and the exact molecular traits involved in the process are not well-characterized. Available evidence point toward critical involvement of the PI3K/AKT/mTOR signaling axis, where mTOR inhibitors may have an important clinical utility (Park et al., 2019).

AST is also observed in *KRAS*-mutant lung cancers upon loss of the *LKB1* tumor suppressor (Han et al., 2014). Genetically engineered mouse models have revealed the mechanistic underpinnings of AST in this molecular context (Chen et al., 2019). Loss of *LKB1* promotes lineage plasticity via a multi-step process that involves remodeling of the extracellular matrix, inactivation of YAP1 and consequent de-repression of the squamous lineage promoting factor, DNp63 (Gao et al., 2014). *LKB1* loss also promotes metabolic reprogramming that leads to an increase in the generation of reactive oxygen species (ROS). Agents that either increase or decrease oxidative stress in the *KRAS* mutant/*LKB1*- model modify AST and thus link redox imbalance to plasticity (Li et al., 2015).

*LKB1* loss is rarely detected in *EGFR*-mutant NSCLC (Koivunen et al., 2008; Gao et al., 2010), but metabolic reprogramming and redox imbalance nonetheless occur and contribute to EGFR TKI resistance (Wang et al., 2019). Chronic, sub lethal EGFR TKI exposure results in increased expression of branched-chain amino acid aminotransferase (BCAT1) via an epigenetically controlled mechanism. When expressed, BCAT1 attenuates ROS production that is required for EGFR TKI-induced cytotoxicity. In metabolically reprogrammed cells, phenethyl isothiocyanate can be used to stimulate ROS production and restore EGFR TKI efficacy. Although the relationship between BCAT1-induced metabolic reprogramming and AST remains to be established, these contemporary studies support the idea that dysregulated metabolic programs may be targeted in *EGFR*-mutant cells to overcome EGFR TKI resistance.

## Conclusion and Remaining Challenges

Advanced *EGFR*-mutant NSCLC can be effectively treated using contemporary EGFR TKIs. However, disease progression is inevitable due to acquired drug resistance. Drug resistance develops by several mechanisms, the least well understood of which is lineage plasticity, that results in both mesenchymal, SCLC/neuroendocrine, and squamous transformation. Routine evaluation of EMT/AST signatures and *TP53/RB1* mutation status in patients newly diagnosed with *EGFR*-mutant LUAD could be implemented to facilitate the early identification of those at risk for phenotypic transformation and aid in treatment planning.

In this review, we present several options to counter drug resistance that results from phenotypic transformation (Table 1). Several questions remain regarding the best therapeutic approach to use. Existing data support an underlying epigenetic basis for lineage plasticity and EMT, SCLC transformation, or AST. This affords an opportunity to intervene with epigenetic therapies. However, several issues will need to be addressed to optimally implement these approaches. First, a number of epigenetic alterations likely co-occur in cells that undergo EMT or SCLC transformation. Indeed, KDM5A, HDAC, and SWI/SNF complexes are all dysregulated in models of EMT-associated EGFR TKI resistance that are available in our labs (unpublished data). It is as yet unclear which epigenetic therapy will prove most efficacious in this context, or whether different individuals will require different interventions. Second, the epigenetic changes that dispose toward SCLC transformation and EMT are rarely studied together and likely to be distinct. Given that *EGFR*-mutant lung tumors are heterogeneous, an individual *EGFR*-mutant lung cancer patient in theory may have subclones that are disposed to EMT and others that are disposed to SCLC. It will therefore be important to understand how a particular epigenetic therapy dynamically influences both processes. Third, the best schedule for administering epigenetic therapy needs to be determined. Epigenetic therapy may be applied early to block lineage plasticity and prevent the emergence of variant phenotypes. If the transformed phenotype has not been fixed, epigenetic therapies may also be used after phenotypic variants appear to restore EGFR dependency and EGFR TKI sensitivity. The benefit of using epigenetic therapy versus taking the alternative approach of targeting the new dependencies of transformed variants (such as FGFR for mesenchymal variants or AURKA for *Rb1*-deficient, transformed SCLC) also needs to be clarified. Ultimately, factors such as tumor heterogeneity, tolerability of combination regimens, patient comorbidities, and accessibility of therapy may all play a role in dictating the right treatment for each patient.

**TABLE 1:** Targets to control phenotypic plasticity in *EGFR*-mutant NSCLC.

**Phenotypic state**	**Acquired dependencies**	**Proposed intervention**
EMT	Epigenetic reprogramming	HDAC*i*, KDM5A*i*, 25(OH)2D3
	Alternative kinase	AXL*i*, IGF1R*i*, FGFR*i*
	Notch-1	Notch-1*i*
	Apoptosis resistance	BH3 mimetics
SCLC	Epigenetic reprogramming	EZH2*i*
	Replicative stress	CHK1*i*, PLK*i*, AURKA*i*
	Alternative kinase	VEGFR*i*, FGFR*i*, c-Kit*i*
	Apoptosis resistance	Bcl-2*i*
SCC	Alternative kinase	mTOR*i*
	Redox imbalance	PEITC

## Author Contributions

TS, LZ, DG, and PH jointly conceived of content. TS and LZ drafted the review, which was edited by DG and PH.

## Conflict of Interest

The authors declare that the research was conducted in the absence of any commercial or financial relationships that could be construed as a potential conflict of interest.

## Abbreviations

ALK: anaplastic lymphoma kinase
ALK-NPM: fusion of ALK with nucleophosmin
AST: adenosquamous transformation
AURK: Aurora kinase
AXL: Axl receptor tyrosine kinase
BCAT1: branched-chain amino acid aminotransferase 1
CDH1: gene that encodes E-cadherin
CHK1: checkpoint kinase 1
EGFR: epidermal growth factor receptor
EMT: epithelial mesenchymal transition
FAK: focal adhesion kinase
FGFR: fibroblast growth factor receptor
HDAC*i*: histone deacetylase inhibitor
IGFR: insulin growth factor receptor
JAK: Janus kinase
LCNEC: large cell neuroendocrine carcinoma
LKB1: serine/threonine kinase 11 or liver kinase B1
LUAD: adenocarcinoma of the lung
MAPK: mitogen-activated protein kinase
MET: MET proto-oncogene
NSCLC: non-small cell lung cancer
PI3K: phosphatidylinositol 3-kinase
PIK3CA: Phosphatidylinositol-4,5-Bisphosphate 3-Kinase Catalytic Subunit Alpha
PLK1: polo like kinase 1
PTEN: phosphatase and tensin homolog
ROS: reactive oxygen species
Rb1: tumor suppressor Retinoblastoma 1
SCC: squamous cell carcinoma
SCLC: small cell lung cancer
STAT: signal transducer and activator of transcription
TP53: tumor protein p53
TKI: tyrosine kinase inhibitor
VEGFR: vascular endothelial growth factor receptor
1,25(OH)2D3: 1,25-dihydroxyvitamin D3.
